# Disseminated Infection Caused by Staphylococcus schleiferi: A Dangerous Wolf in Coagulase-Negative Staphylococcus Clothing

**DOI:** 10.7759/cureus.11188

**Published:** 2020-10-26

**Authors:** Seioh Ezaki, Hiroshi Ito, Yasuhiro Ogawa, Nobutake Shimojo, Satoru Kawano

**Affiliations:** 1 Orthopaedics, University of Tsukuba Hospital, Tsukuba, JPN; 2 Division of Hospital Medicine, University of Tsukuba Hospital, Tsukuba, JPN

**Keywords:** staphylococcus schleiferi, infective endocarditis, brain abscesses, bacteremia

## Abstract

*Staphylococcus schleiferi* is a coagulase-negative staphylococcus known to cause canine external otitis but has rarely been reported in human infections. However, unlike other coagulase-negative staphylococci, *S. schleiferi* can cause disseminated infection in immunocompetent patients. Here, we present a case of *S. schleiferi* bacteremia, accompanied by infective endocarditis, brain abscesses, acute focal bacterial nephritis, and possible epididymitis, in which an *S. aureus* bacteremia treatment strategy was useful for resolution. Further reports should be accumulated to determine if *S. schleiferi* is a virulent pathogen that frequently causes the disseminated infection type seen in our patient.

## Introduction

*Staphylococcus schleiferi* is a coagulase-negative staphylococcus that has rarely been reported in human infections [1]. Described reports of infection are mainly limited to canine external otitis [2] and little is known about its pathogenicity and presentation in human infections. Here, we report a patient with brain abscesses caused by *S. schleiferi* that presented as a disseminated infection and a treatment strategy for *S. aureus* bacteremia [3] was useful in managing this condition.

## Case presentation

**Figure 1:**
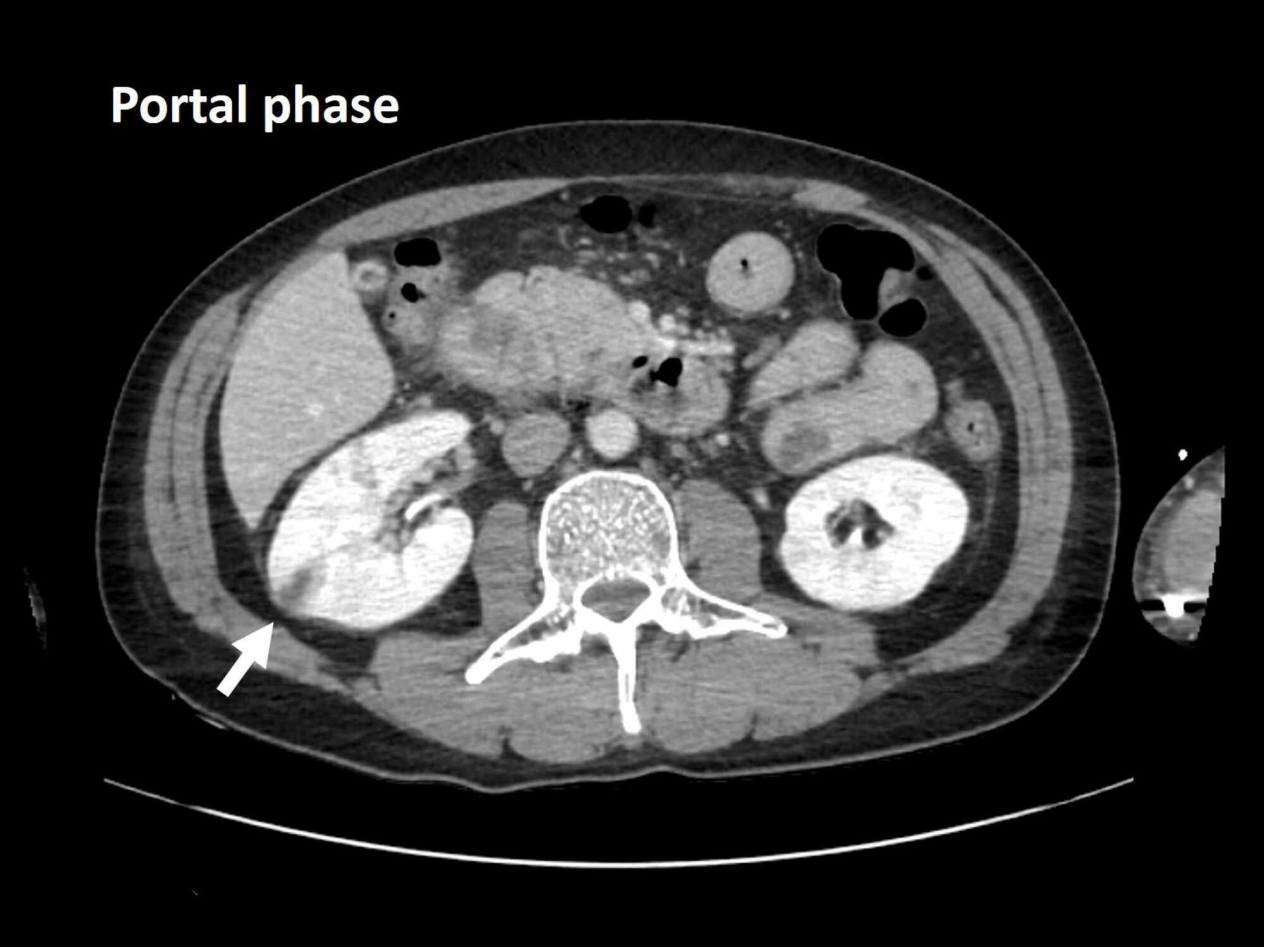
Initial contrast-enhanced computed tomography images of the chest and abdomen The computed tomography images showing a wedge-shaped area in the right kidney (white arrow).

**Figure 2:**
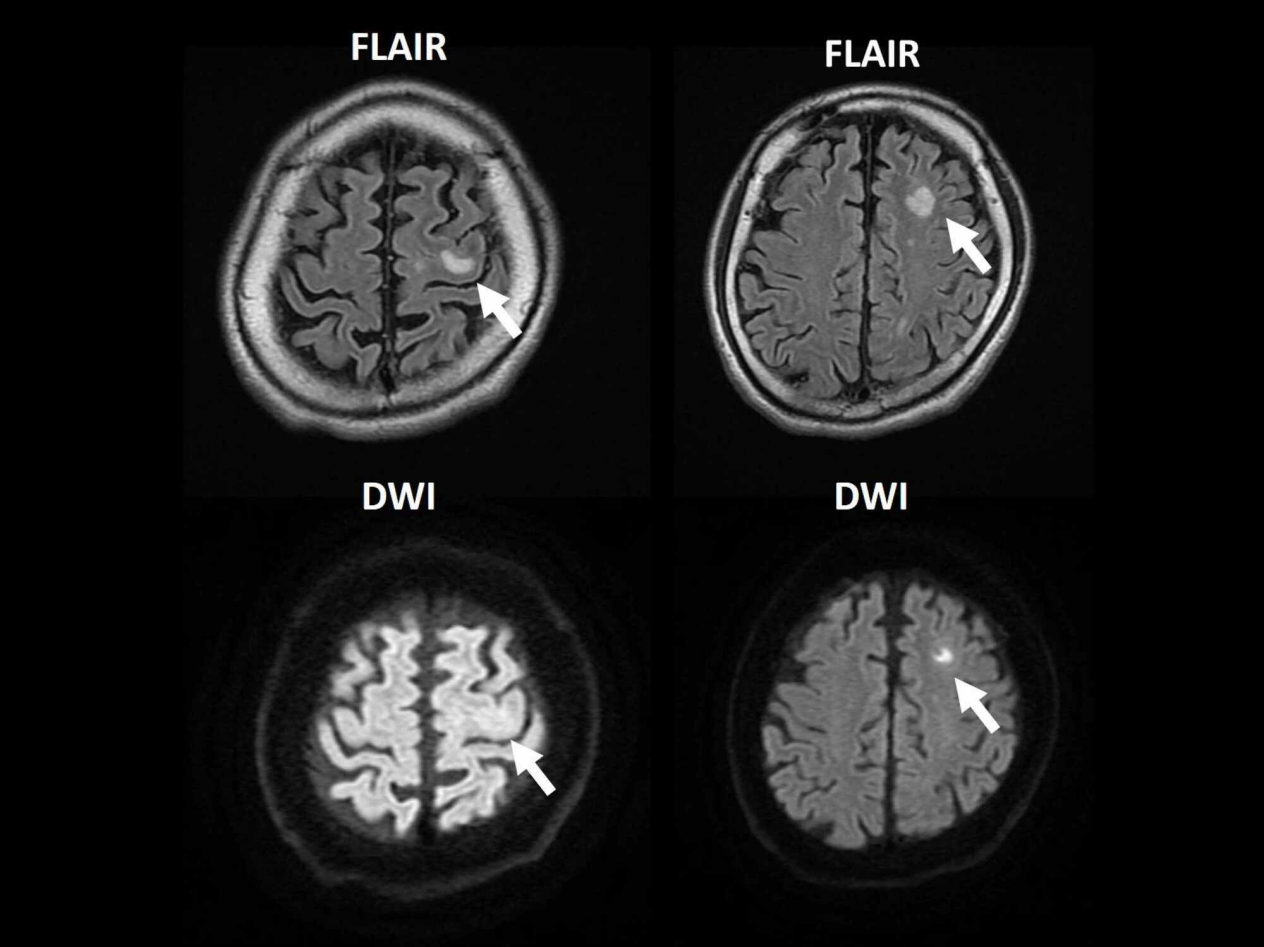
Initial magnetic resonance images of the brain The magnetic resonance images showing scattered abnormal signal intensities in the left parietal and frontal lobes (white arrows).

**Figure 3:**
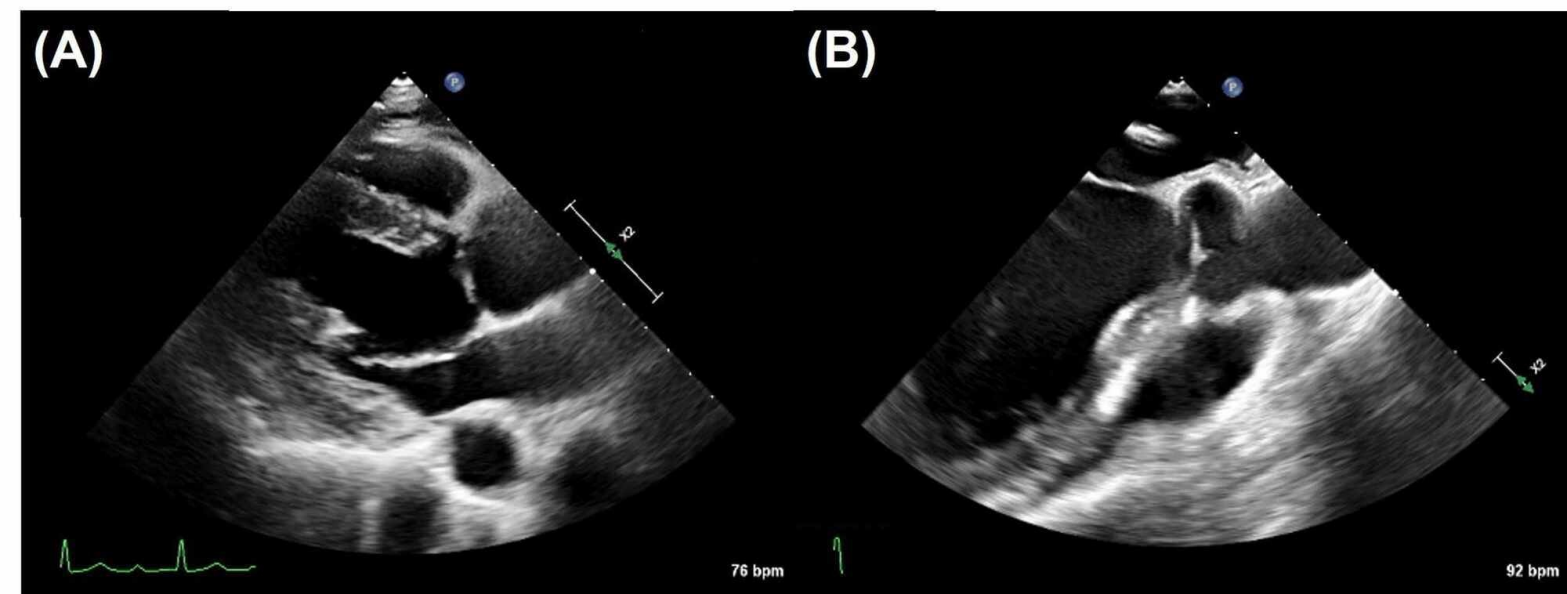
Transthoracic and transesophageal echocardiography Transthoracic (A) and transesophageal echocardiography (B) revealed no remarkable abnormalities suggesting infective endocarditis.

A 45-year-old Japanese man was admitted to the emergency department after seven days of persistent fever, nausea, and right scrotal pain. His medical history included aortic valve regurgitation due to a bicuspid aortic valve (native valve) and a dental extraction without antibiotic prophylaxis one week before the onset of symptoms. He had owned a dog until two years before. He denied recent sexual history. His heart rate was 115 beats per minute, blood pressure 141/83 mmHg, axillary temperature 37.8 ℃ and respiratory rate 23. Physical examination was unremarkable except for right scrotal tenderness. Laboratory data were as follows: white blood cell count 11,900/μL (reference value, 4,000-9,000/μL), hemoglobin 15.3 g/dL (reference value, 14.0-18.0 g/dL), platelet count 246,000/μL (reference value, 150,000-350,000/μL), aspartate transaminase 114 U/L (reference value, 8-38 U/L), alanine aminotransferase 193 U/L (reference value, 4-44 U/L), lactate dehydrogenase 318 U/L (reference value, 124-222 U/L), serum creatinine 0.69 mg/dL (reference value, 0.61-1.04 mg/dL), and C-reactive protein 10.9 mg/dL (reference value, 0.0-0.2 mg/dL). Contrast-enhanced computed tomography images of the chest and abdomen revealed a wedge-shaped area in the right kidney (Figure 1) while magnetic resonance (MR) images of the brain showed scattered abnormal signal intensities in the left parietal and frontal lobes (Figure 2). Native-valve infective endocarditis was suspected; however, transthoracic and transesophageal echocardiography revealed no remarkable abnormalities except for the previously known aortic valve regurgitation (Figure 3). As antistaphylococcal penicillins are not available in Japan, high-dose ceftriaxone (2 g, every 12 hours) was administered for presumed disseminated bacterial infection by the dental extraction and blood cultures revealed gram-positive cocci in clusters which were identified as methicillin-susceptible *S. schleiferi* (Table 1).

**Table 1:** Antimicrobial susceptibility of S. schleiferi isolated from blood cultures MIC: minimum inhibitory concentration, ST: sulfamethoxazole-trimethoprim

Antibiotics	MIC (μg/mL)	Sensitivity
Benzylpenicillin	>8	Resistant
Methicillin	≤0.25	Sensitive
Cefazolin	≤8	Sensitive
Cefotiam	≤8	Sensitive
Cefepime	≤8	Sensitive
Cefmetazole	≤16	Sensitive
Meropenem	≤4	Sensitive
Minocycline	≤4	Sensitive
Gentamicin	>8	Resistant
Clindamycin	≤0.5	Sensitive
Levofloxacin	>4	Resistant
Vancomycin	≤2	Sensitive
Linezolid	4	Sensitive
ST	≤2	Sensitive
Rifampicin	≤1	Sensitive

**Figure 4:**
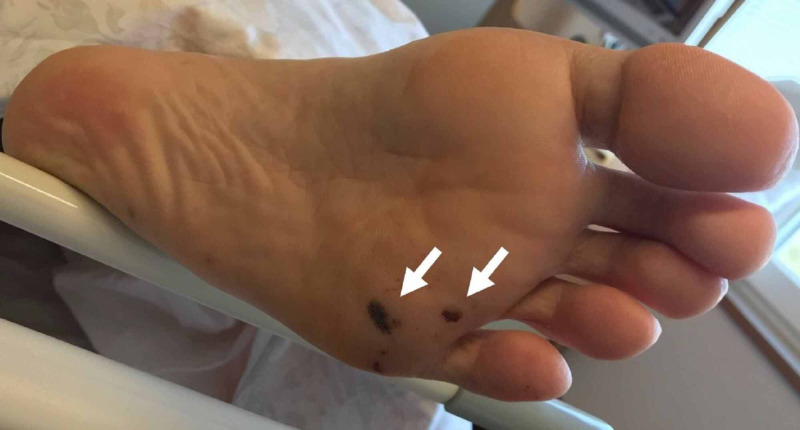
Osler’s nodules on his left sole Tender nodules on the left sole as seen on the eighth hospital day (white arrows).

His symptoms resolved within several days using high-dose ceftriaxone and two sets of repeated blood cultures were negative. On the eighth hospital day, tender nodules developed on his left sole, which were thought to be Osler’s nodules (Figure 4), and a subsequent diagnosis of infective endocarditis was made according to the modified Duke criteria [4]. The abnormal signal intensity on the brain MR images improved on day 39 and he was discharged after a 42-day course of ceftriaxone therapy. At his most recent visit, 30 days after discharge, he was asymptomatic and doing well.

## Discussion

We presented a case of disseminated infection caused by *S. schleiferi*, a relatively rare coagulase-negative staphylococcus. Coagulase-negative staphylococci are considered less virulent than *S. aureus* [5]; however, there have been reports of *S. schleiferi* causing severe disease in cases of infective endocarditis [6,7], pericarditis [8], prostate abscess [9], meningitis [10], and vertebral osteomyelitis [11] (Table 2). Our patient presented with not only bacteremia but also brain abscesses and acute focal bacterial nephritis in the right kidney. He was additionally diagnosed with infective endocarditis according to the modified Duke criteria for which he met five minor clinical criteria: bicuspid aortic valve, fever, intracranial hemorrhage, Osler’s nodes, and positive blood cultures which did not meet a major criterion. Physicians should bear in mind *S. schleiferi* bacteremia can cause disseminated infection.

**Table 2:** Previous reports on S. schleiferi bacteremia CTRX: ceftriaxone, TOB: tobramycin, PCG: benzylpenicillin, RFP: rifampicin, GM: gentamicin, VCM: vancomycin, AZT: aztreonam, CEZ: cefazolin, MEPM: meropenem

Case	Author	Age (years)	Gender	Underlying disease	Focus	Antibiotics	Prognosis
1	Latorre M, et al. [1]	66	Male	Cirrhosis	Unknown	CTRX, TOB	Survived
2	Leung MJ, et al. [6]	78	Male	Myxomatous mitral valve (post prosthetic valve replacement), atrial fibrillation	Infective endocarditis	PCG, RFP, GM	Survived
3	Kumar D, et al. [7]	58	Male	Chronic hepatitis C (post liver transplantation)	Infective endocarditis	VCM, GM	Survived
4	Thawabi M, et al. [8]	55	Male	None	Infective pericarditis	VCM, AZT	Survived
5	Merchant C, et al. [9]	49	Male	Diabetes mellitus, prostatic hyperplasia	Prostate abscess	CEZ, VCM	Survived
6	Jin D, et al. [10]	0	Male	None	Meningitis	VCM, MEPM, CTRX	Survived
7	Yarbrough ML, et al. [11]	60	Female	None	Vertebral osteomyelitis	VCM, CTRX	Survived
8	Our case	45	Male	Bicuspid aortic valve (native valve)	Brain abscess, focal nephritis, epididymitis	CTRX	Survived

An *S. aureus* bacteremia treatment strategy, including follow-up blood cultures, early source control, echocardiography, early use of appropriate antibiotics (although antistaphylococcal penicillins are unavailable in Japan), and optimal treatment duration, was useful in resolving this condition [12]. As our patient had brain abscesses complicated by infective endocarditis, we used a 42-day course of intravenous antibiotic therapy in line with a previous report [13]. Surgical intervention was deemed unnecessary in our case since the brain abscesses were relatively small and transthoracic/transesophageal echocardiography revealed no remarkable findings suggestive of verrucous endocarditis.

The occurrence of severe infections caused by coagulase-negative staphylococci is not limited to *S. schleiferi* bacteremia as *S. lugdunensis* is well known to cause severe diseases, such as infective endocarditis [14]. Bacteremia caused by these pathogens should thus be treated as *S. aureus* bacteremia instead of as other coagulase-negative staphylococci [15].

## Conclusions

We presented a case of *S. schleiferi* bacteremia accompanied by infective endocarditis, brain abscesses, acute focal bacterial nephritis, and possible epididymitis. *S. schleiferi* may present as a disseminated infection, as seen in our case, but can be effectively managed according to *S. aureus* bacteremia guidelines. Further reports should be accumulated to determine if *S. schleiferi* is a highly virulent pathogen that frequently causes disseminated infection types as seen in our case.
